# Actin Bundle Nanomechanics and Organization Are Modulated by Macromolecular Crowding and Electrostatic Interactions

**DOI:** 10.3389/fmolb.2021.760950

**Published:** 2021-11-26

**Authors:** Nicholas Castaneda, Cecile Feuillie, Michael Molinari, Ellen Hyeran Kang

**Affiliations:** ^1^ NanoScience Technology Center, University of Central Florida, Orlando, FL, United States; ^2^ Burnett School of Biomedical Sciences, College of Medicine, University of Central Florida, Orlando, FL, United States; ^3^ Institute of Chemistry and Biology of Membranes and Nano-objects, CBMN CNRS UMR 5248, IPB, Université de Bordeaux, Pessac, France; ^4^ Department of Physics, University of Central Florida, Orlando, FL, United States; ^5^ Department of Materials Science and Engineering, University of Central Florida, Orlando, FL, United States

**Keywords:** actin bundles, macromolecular crowding, cations, nanomechanics, atomic force microscopy

## Abstract

The structural and mechanical properties of actin bundles are essential to eukaryotic cells, aiding in cell motility and mechanical support of the plasma membrane. Bundle formation occurs in crowded intracellular environments composed of various ions and macromolecules. Although the roles of cations and macromolecular crowding in the mechanics and organization of actin bundles have been independently established, how changing both intracellular environmental conditions influence bundle mechanics at the nanoscale has yet to be established. Here we investigate how electrostatics and depletion interactions modulate the relative Young’s modulus and height of actin bundles using atomic force microscopy. Our results demonstrate that cation- and depletion-induced bundles display an overall reduction of relative Young’s modulus depending on either cation or crowding concentrations. Furthermore, we directly measure changes to cation- and depletion-induced bundle height, indicating that bundles experience alterations to filament packing supporting the reduction to relative Young’s modulus. Taken together, our work suggests that electrostatic and depletion interactions may act counteractively, impacting actin bundle nanomechanics and organization.

## Introduction

Actin bundles form critical cytoskeletal assemblies, such as filopodia (Mogilner and Rubinstein, 2005; Blanchoin et al., 2014) and stress fibers (Naumanen et al., 2008), and facilitate cellular mechanosensing (Colombelli et al., 2009; Narayanan et al., 2015; Rückerl et al., 2017). Actin bundle formation occurs in intracellular environments that are crowded with various ions and macromolecules (Ellis, 2001; Minton, 2001; Sarkar et al., 2013; Kuznetsova et al., 2014). To generate actin bundles, the lateral aggregation of actin filaments is overcome through counterion condensation (Manning, 1978; Angelini et al., 2003; Angelini et al., 2005; Castaneda et al., 2018) or depletion interactions (Hosek and Tang, 2004; Ping et al., 2006; Tharmann et al., 2006; Sukenik et al., 2013; Sapir and Harries, 2014). These environmental factors promote actin bundling through either electrostatic interactions reducing the surface charges on actin filaments (Tang and Janmey, 1996; Angelini et al., 2003; Huber et al., 2012; Castaneda et al., 2018) or steric exclusion and/or non-specific (enthalpic) effects (Hosek and Tang, 2004; Streichfuss et al., 2011; Huber et al., 2015).

Both cation interactions and macromolecular crowding can modulate the mechanical and structural properties of actin bundles (Tang and Janmey, 1996; Angelini et al., 2003; Hosek and Tang, 2004; Angelini et al., 2005; Tharmann et al., 2006; Fazli et al., 2009; Gao et al., 2015; Castaneda et al., 2018). Divalent cations (Mg^2+^ and Ca^2+^) were shown to condense actin filaments into bundles, with bending persistence lengths ranging from ∼15 to 45 µm (Castaneda et al., 2018), and promote overtwisting of the bundle structure (Angelini et al., 2003). The stiffness and elasticity of cation-induced actin bundle networks increased with increasing (Mg^2+^) (Gurmessa et al., 2019). In comparison, the bending stiffness (Claessens et al., 2006) and elastic moduli (Tharmann et al., 2006) of depletion-induced bundles correlated with increasing concentrations of macromolecular crowding. Depletion-induced bundles can exhibit enhanced mechanical properties, allowing for minimal bending deformation in response to external forces (Martiel et al., 2020). Although these studies focused on investigating actin bundling on the macroscale, how macromolecular crowding and electrostatic interactions influence actin bundle mechanics and structure on the nanoscale is not well established.

Atomic force microscopy (AFM) is a useful tool in identifying and quantifying nanoscale mechanical and structural changes to actin cytoskeleton *in vitro* or in cells (Sharma et al., 2010; Calzado-Martín et al., 2016; Usukura et al., 2016; Liu et al., 2020). AFM recently revealed the detailed organization of actin filaments and bundles beneath cellular membranes (Usukura et al., 2016; Liu et al., 2020). Conformational changes in drebrin-bound filaments have been demonstrated by high-resolution AFM imaging (Sharma et al., 2010). Furthermore, a recent AFM study investigated the changes to relative Young’s modulus of actin stress fibers in epithelial breast cancer cells (Calzado-Martín et al., 2016). However, knowledge on changes to actin bundle mechanics and organization *in vitro* under varying intracellular environmental conditions is lacking.

In this study, we demonstrate the influence of electrostatic and depletion interactions on the mechanics and organization of cation- or depletion-induced actin bundles. We hypothesize that combined environmental factors drive alterations to actin bundle relative Young’s modulus ( E ) and organization on the nanoscale. To determine bundle nanomechanics, we used high-resolution AFM to visualize and reveal changes in bundle height as well as perform nanoindentation measurements. We demonstrate that varying macromolecular crowding or cation conditions leads to a significant modulation in bundle E as well as packing. Overall, this work implicates that the electrostatic and depletion interactions can act counteractively on actin bundling, modulating actin cytoskeleton mechanics and structure *in vivo*.

## Materials and Methods

### Sample Preparation

Purification of actin monomers (G-actin) from rabbit skeletal muscle acetone powder (PelFreeze Biologicals Inc., Rogers, AR, USA) was performed through gel filtering G-actin over Sephacryl S300 size exclusion column equilibrated in buffer A (0.2 mM CaCl_2_, 1 mM NaN_3_, 2 mM Tris-HCl pH 8.0, 0.2 mM ATP, and 0.5 mM DTT) as previously described (Kang et al., 2012; Castaneda et al., 2018; Castaneda et al., 2019; Heidings et al., 2020). G-actin bound with Ca^2+^ was subjected to cation exchange by ethylene glycol-bis(β-aminoethyl ether)-N,N,N,N-tetraacetic acid (EGTA) to Mg^2+^ with the addition of 0.2 mM EGTA and MgCl_2_ concentration equal to the initial G-actin concentration plus 10 μM. Following the cation exchange, polymerization of G-to actin filaments (F-actin) was performed (Kang et al., 2012; Castaneda et al., 2018; Castaneda et al., 2019; Heidings et al., 2020).

To form actin bundles, unlabeled F-actin was allowed to polymerize for 1 to 2 h and subjected to high concentrations of either cations or crowding agents. Cation-induced bundles were formed by the use of 0.1 volume 10X MI buffer (300 mM Mg^2+^, 100 mM imidazole, pH 7.0, 10 mM ATP, and 10 mM DTT). Formation of depletion-induced bundles was achieved by the addition of crowding buffer [Ficoll 70 20% w/w or polyethylene glycol (PEG) 5% w/w] in 1X KMI (50 mM KCl, 2 mM Mg^2+^, 10 mM imidazole pH 7.0, 1 mM ATP, and 1 mM DTT). The crowding agent concentrations for the experiments were Ficoll 70 (Thermo Fisher Scientific Inc., Waltham, MA, USA), 1% w/w (∼0.16 mM), 5% w/w (∼0.79 mM), and 10% w/w (∼1.56 mM), or PEG, 8 kDa (Thermo Fisher Scientific Inc., Waltham, MA, USA), at 1% w/w (∼1.38 mM), 5% w/w (∼6.94 mM), and 10% w/w (∼13.88 mM). These specific crowding conditions were chosen based on the intracellular volume occupancy of ∼5–40% (Ellis and Minton, 2003; Kuznetsova et al., 2014), while (Mg^2+^) (10–50 mM) was selected based on previous work shown in Castaneda *et al*. (2018) and intracellular (Mg^2+^) (Romani, 2011).

### Atomic Force Microscopy Imaging and Nanomechanics Analysis

Mica substrates were freshly cleaved, and the addition of 50 μl of positively charged binding agent (3-aminopropyl)triethoxysilane (APTES) (0.1% v/v) was done by pipetting onto the mica surface and allowing to bind for 10 min (Liu et al., 2005; Lyubchenko, 2011) to favor actin bundle adhesion on the substrate. Prior to the addition of bundle samples, the APTES-coated mica was rinsed with a gentle stream of ddH_2_O and dried with compressed air (Liu et al., 2005; Lyubchenko, 2011). AFM experiments were performed on the coated substrates to check their topographical and mechanical homogeneities and their low rugosity as shown in Supplementary Figure S1. Following the drying of the mica surface, 5 μl of the bundle sample in the respective crowding or cation environment, at a concentration of ∼10 μM, was placed on top the APTES-coated mica surface and allowed to bind for ∼5 min (Liu et al., 2005). Then, a corresponding sample buffer was added onto the bundle-bound mica surface at a volume of ∼50 μl.

The nanomechanical and height changes of the bundles in the liquid environment were determined using a Dimension FastScan AFM (Bruker, Santa Barbara, CA, USA) at a constant temperature of 20°C. Imaging was first conducted in Peak Force Quantitative Nanomechanical Mapping (PFQNM) tapping mode (256 pixels × 256 pixels) at a scan size ranging from 1 to 5 μm. The AFM cantilever tips used for the experiments were gold-coated FASTSCAN-C tips (Bruker, Santa Barbara, CA, USA) with a triangular tip shape, calibrated tip radius of ∼5 nm, nominal spring constant of 0.8 N/m, and nominal resonant frequency of ∼300 kHz. The nanomechanical experiments were performed by force curve measurements after proper calibration of the setup. Cantilever tips were calibrated as previously described in Heu *et al*. (2012). Briefly, prior to each measurement, the deflection sensitivity (by capturing three force curves on a non-compliant part of the sample and averaging the three corresponding slopes on the linear portions of the curves) and the spring constant (by tuning the cantilever at least 10 µm away from the surface and spotting the resonance peak) were calculated for each probe in dilute, crowded, or cation buffer conditions. The tip radius for each probe was determined before and after experiments using a reference titanium roughness sample (TipCheck Sample, Bruker, USA). A PeakForce frequency of 0.25 kHz was used in order to maximize the contact time between the tip and the sample, with a PeakForce amplitude of ∼1 µm. The loading force was adjusted to 800 pN. A small approach velocity of 6 μm/s was used to minimize the contribution of viscosity to the mechanical response. Thus, the hydrodynamic damping hysteresis was almost suppressed and had no impact on the relative Young’s modulus measurements. For the determination of the relative Young’s modulus, the retraction curves were used in response to the retraction and the approach curves showing a similar negligible contribution of viscosity for cation- and depletion-induced bundles in crowding and cation environments. Regarding the geometry of the tip and the negligible adhesion in the force-curve, the relative Young’s modulus was determined using a classic Hertz model to fit the force curves:

$$F=\;\frac{4}{3}\;\frac{E}{\left(1-v^{2}\right)}\sqrt{R}\delta^{3/2}$$ (1)

where E is the relative Young’s modulus of the actin bundle, v is the estimated Poisson ratio of the bundle, R is the nominal radius of the tip, and δ is the indentation depth. The Poisson ratio of actin has been previously investigated to be ∼0.3 for actin filaments and crosslinked bundles (Tseng et al., 2002; Lin et al., 2017; Wang et al., 2020). We estimated our E values with an assumed Poisson value of 0.3. For each condition, at least 150 curves were analyzed. Analysis of the collected force curve bundle measurements was achieved by the use of NanoScope Analysis v. 2.0 software (Bruker). Alterations to the height of individual bundles in crowded or cation environments were analyzed by the profile extracting tool in Gwyddion software (Nečas and Klapetek, 2012).

### Statistical Analysis

The statistical significance for each of the actin bundle E and the height measurements were determined using OriginLab v.8.5 software. Multiple analysis of variance and *post-hoc* Tukey test determined the probability (*p*-value) showing the significant modulations between samples (notations for *p*-values: n.s., not significant, *p < 0.05, **p < 0.01, ***p < 0.001).

## Results

### Cation-Induced Bundle Height and Relative Young’s Modulus Are Modulated in Crowded Environments

**FIGURE 1:**
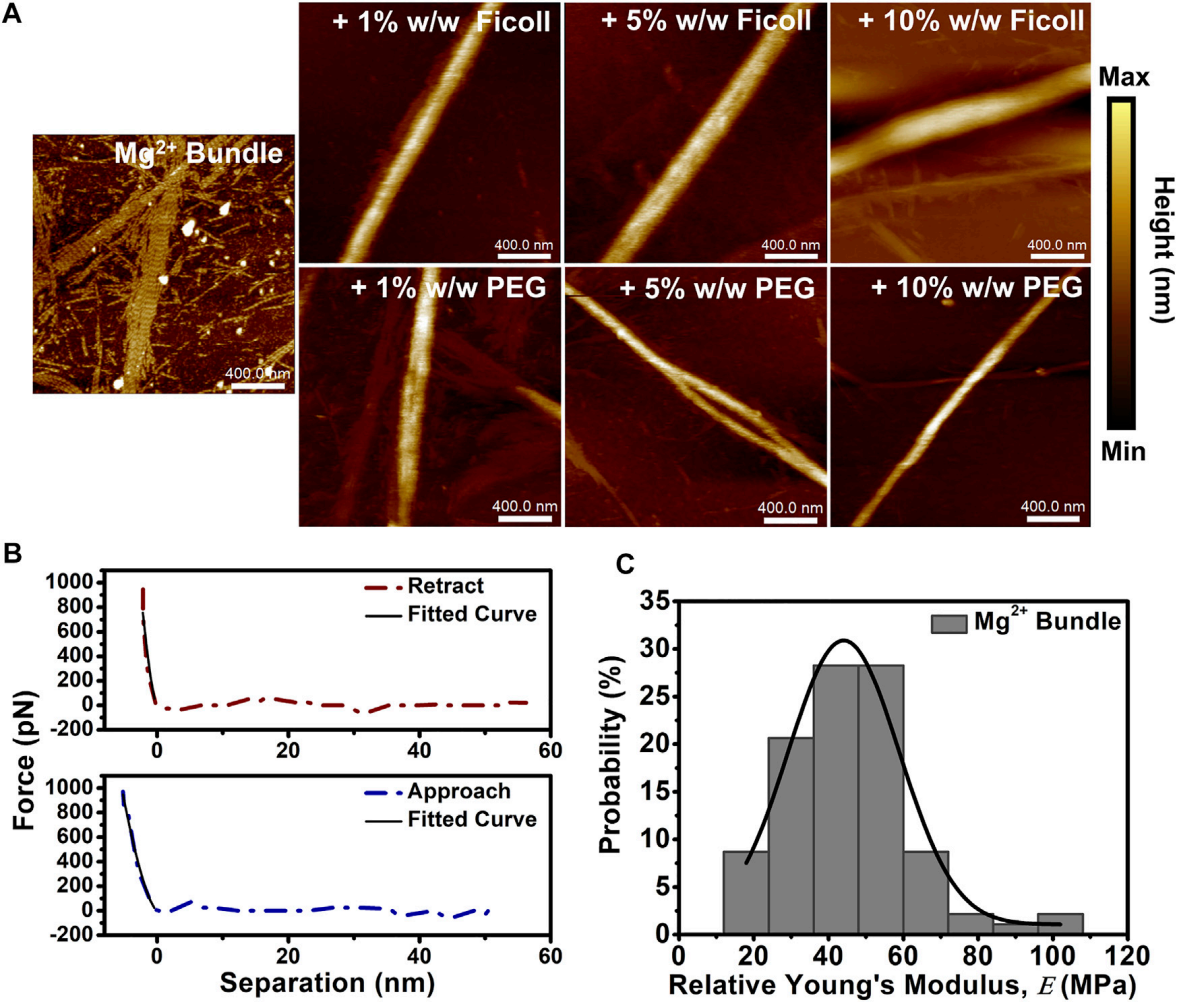
Cation-induced bundle atomic force microscopy (AFM) imaging and nanomechanical analysis in macromolecular crowded environments. **(A)** Representative AFM images of cation-induced bundles in the absence and presence of increasing concentrations of Ficoll (1–10% w/w) or PEG (1–10% w/w). Actin bundle ≈ 15 µM and scale bar = 400 nm. **(B)** Representative cation-induced actin bundle retract and approach force curves demonstrating the elastic behavior of the bundle; solid lines indicate fit using Hertz model. **(C)** Histogram of Mg^2+^-induced bundle control E obtained from force curve measurements along the bundle. The distribution of E is fit with Gaussian function (solid line). Total number of force curves analyzed *N* ≈ 150.

We used AFM to determine the height and nanomechanical properties of cation-induced actin bundles in the solution of macromolecular crowding agents. Cation-induced bundles were formed by the addition of physiologically relevant divalent cation (10–50 mM Mg^2+^), and then bundles were placed in buffer solutions with varying concentrations of Ficoll or PEG (1–10% w/w). Dilute buffer conditions displayed a mixture of both cation-induced actin bundles and actin filaments (Figure 1A). Furthermore, cation-induced bundle control exhibited short periodic striations along the bundle surface not visualized with the addition of Ficoll or PEG (Figure 1A). Upon addition of Ficoll and PEG (1–10% w/w), the bundles exhibited an increase in height (Figure 1A). Bundle control displayed a height distribution centered at ∼8 nm; however, bundle height was shown to increase with a shift in the distribution center to ∼30 nm in Ficoll (10% w/w) (Supplementary Figure S2A). In addition, cation-induced bundles in varying PEG environments were similarly shown to have a distribution centered at ∼8 nm (Supplementary Figure S2B). When increasing the concentration of PEG to 10% w/w, the bundle height distribution centered at ∼20 nm; however, bundle heights of ∼45 nm can occasionally be observed (Supplementary Figure S2B).

**FIGURE 2:**
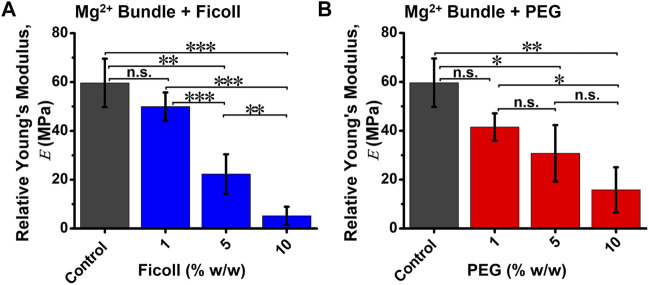
The relative Young’s modulus (*E*) of cation-induced bundle in the absence and presence of varying macromolecular crowded environments. Quantification of average bundle E in **(A)** Ficoll (1–10% w/w) or **(B)** PEG (1–10% w/w) conditions. Cation-induced bundle E was fitted using Hertz model, and significant difference to bundles was determined by Tukey test (n.s., not significant; *p < 0.05, **p < 0.01, ***p < 0.001). Total number of force curves analyzed per condition *N* ≈ 150, and error bars indicate standard deviation.

The nanomechanical properties of bundles, such as relative Young’s modulus ( E ), can be obtained from the force curve measurements provided by PFQNM mode for each pixel of the obtained image. Force curve measurements were taken along the center of cation-induced bundles and analyzed for changes to E (Figure 1B). Interestingly, histograms of cation-induced bundle E in crowding revealed that increasing Ficoll and PEG concentrations narrow and shift the distribution to reduced E values (Supplementary Figure S3). The cation-induced bundle control showed an averaged value of E ∼60 ± 9.9 MPa (Figure 2A). The addition of the lowest Ficoll condition (1% w/w) leads to ∼8% reduction in bundle E (∼49 ± 5.8 MPa) (Figure 2A). However, increasing Ficoll up to 10% w/w, the bundles exhibit a significant decrease in E by approximately tenfold (∼5.0 ± 3.7 MPa) (Figure 2A). For cation-induced bundles in PEG, the results showed a more drastic reduction in bundle E with the initial presence of PEG (1% w/w), reducing bundle E by ∼45% (∼32 ± 17 MPa) (Figure 2B). In addition, as the PEG concentration was increased to 10% w/w, a reduction of approximately fourfold in bundle E , with a value of ∼15 ± 9.2 MPa, was determined, as compared to bundle control (∼60 MPa) (Figure 2B).

### Depletion-Induced Bundle Height and Relative Young’s Modulus Are Influenced by Cation Environments

**FIGURE 3:**
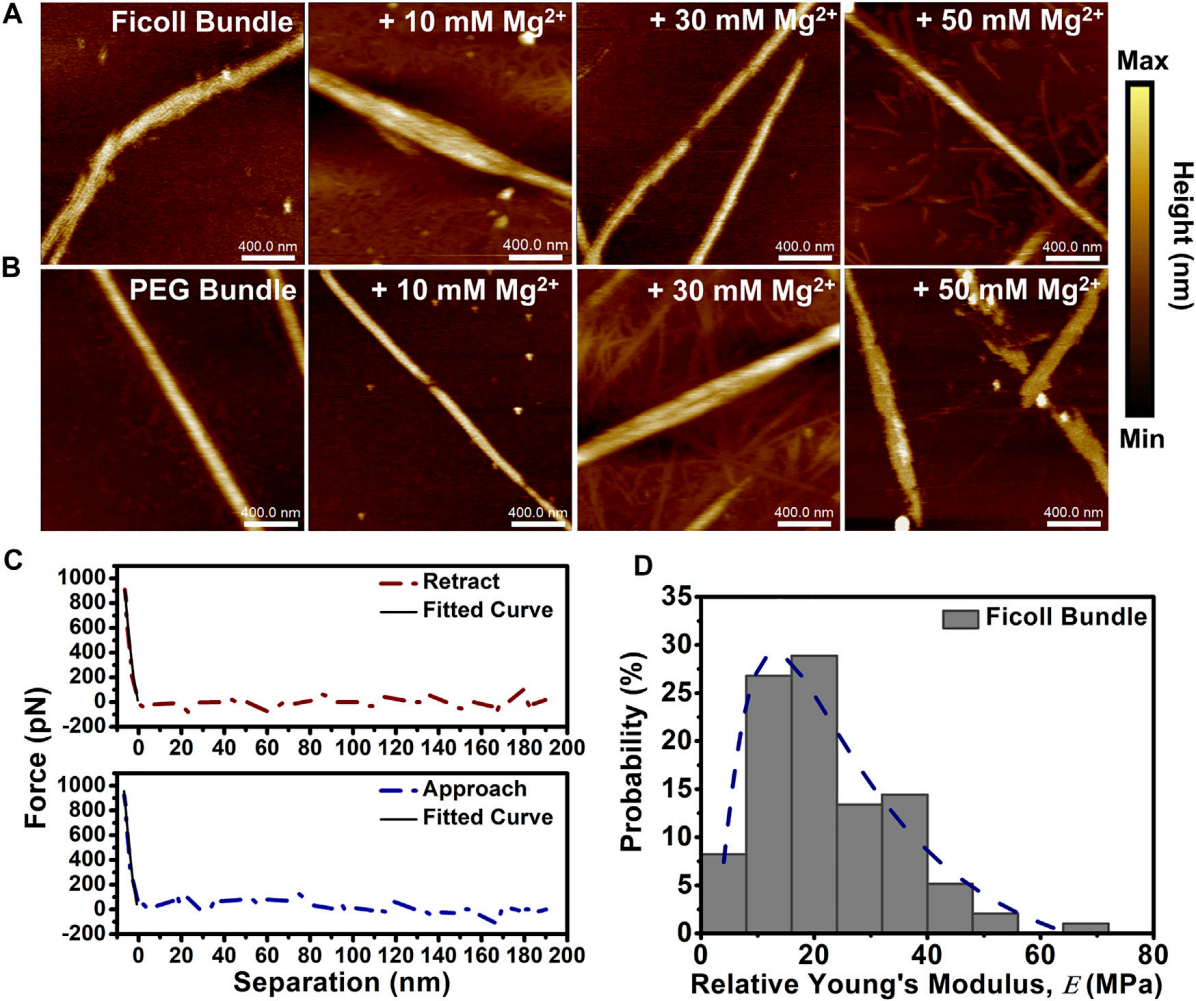
Depletion-induced actin bundle atomic force microscopy (AFM) imaging and nanomechanical analysis. **(A**, **B)** Representative AFM images of depletion-induced bundles in the absence and presence of increasing (Mg^2+^) (10–50 mM Mg^2+^). **(A)** 20% w/w Ficoll bundles + 10–50 mM Mg^2+^ and **(B)** 5% w/w PEG bundles + 10–50 mM Mg^2+^. Actin bundle ≈ 15 µM and scale bar = 400 nm. **(C)** Representative Ficoll-induced actin bundle retract and approach force curves demonstrating the elastic behavior of the bundle; solid lines indicate fit using Hertz model. **(D)** Histogram of Ficoll-induced bundle control E acquired from force curve measurements along the bundle. The distribution of E is fit with log-normal function (dashed line). Total number of force curves analyzed *N* ≈ 150.

We set out to determine the height and nanomechanical changes to depletion-induced bundles in solutions with varying (Mg^2+^) using AFM. We formed bundles by the addition of crowding agents Ficoll (20% w/w) or PEG (5% w/w) and subjected the bundles to divalent cation (Mg^2+^) (10–50 mM). Both Ficoll- and PEG-induced actin bundle controls did not visibly display short periodic striations as previously shown in cation-induced bundles, possibly due to the initial presence of crowding agents (Figures 3A,B). The Ficoll-induced actin bundle height was shown to increase at 50 mM Mg^2+^, while the PEG-induced bundle height increased at both 30 and 50 mM Mg^2+^ (Figures 3A,B). The Ficoll-induced bundle control height was shown to center at ∼7 nm and showed a broadening of height distribution at the lowest 10 mM Mg^2+^ (Supplementary Figure S4A). At 30 mM Mg^2+^, bundle height distribution was shown to be mainly centered at ∼8 nm, while 50 mM Mg^2+^ bundle height distribution is shown to center at ∼14 nm (Supplementary Figure S4A). The PEG-induced bundle control height was measured to be distributed at ∼10 nm (Supplementary Figure S4B). However, increasing (Mg^2+^) led to significant alterations in PEG-induced bundle height observed at 30 mM (Mg^2+^), with a distribution centered at ∼30 nm and with occasional bundles observed at ∼60 nm (Supplementary Figure S4B). Upon increasing the (Mg^2+^) to 50 mM, the bundle height shifts in distribution to ∼14 nm (Supplementary Figure S4B).

**FIGURE 4:**
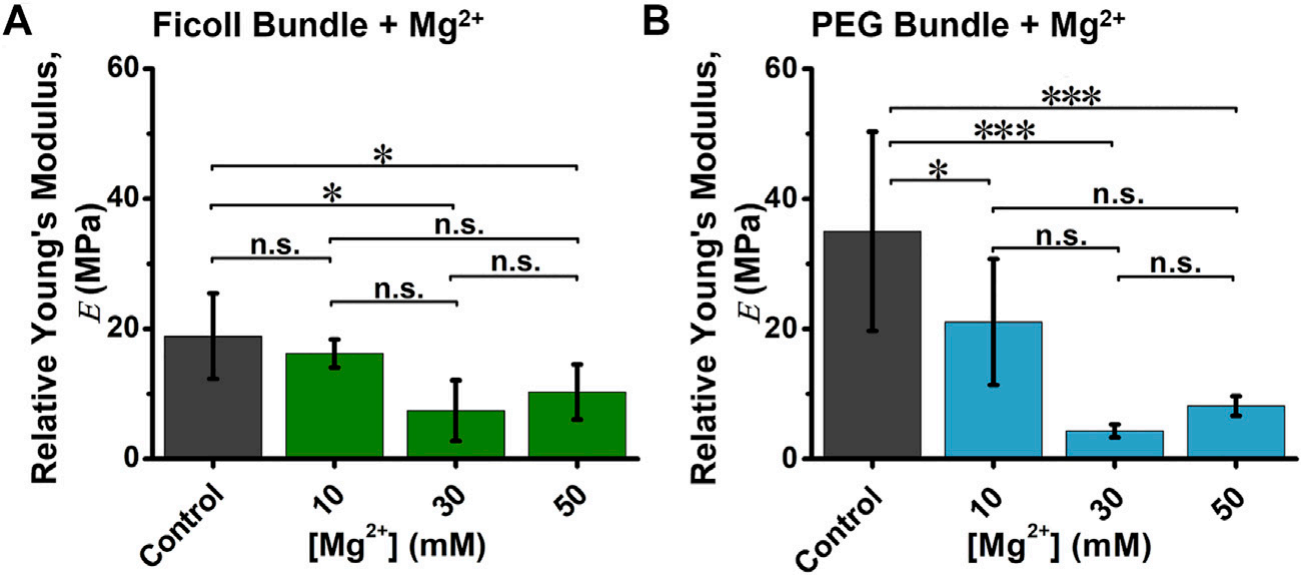
The relative Young’s modulus (*E*) of depletion-induced bundle in the absence and presence of varying (Mg^2+^). Quantification of average **(A)** Ficoll-induced or **(B)** PEG-induced actin bundle E in 10–50 mM (Mg^2+^). Depletion-induced bundle E was fitted using Hertz model, and significant difference to bundles was determined by Tukey test (n.s., not significant; *p < 0.05, **p < 0.01, ***p < 0.001). Total number of force curves analyzed per condition *N* ≈ 150, and error bars indicate standard deviation.

Next, we analyzed the relative Young’s modulus ( E ) of depletion-induced actin bundles in varying (Mg^2+^) (10–50 mM) (Figure 3C). We collected and analyzed force curve measurements to determine the variations of depletion-induced bundles E as previously performed with cation-induced bundles. The histograms of Ficoll-induced actin bundles demonstrated that the bundles experience a narrowing in overall distribution and a shift to lower values of E as the (Mg^2+^) increases (Supplementary Figure S5A). In addition, PEG-induced bundles showed a similar behavior of narrowing and reduced E values with rising (Mg^2+^) (Supplementary Figure S5B). The Ficoll-induced bundle control showed an average of E ∼18 ± 6.6 MPa (Figure 4A). The addition of initial (Mg^2+^) (10 mM) demonstrated a slight reduction in Ficoll-induced bundle E , with a reduction of ∼10% (∼16 ± 2.2 MPa) (Figure 4A). However, increasing the (Mg^2+^) up to 30 mM showed the greatest change with bundle E ∼7 ± 4 MPa, an approximately twofold reduction (Figure 4A). On the other hand, the PEG-induced bundle control E showed an average of ∼35 ± 15 MPa (Figure 4B). At the lowest (Mg^2+^) (10 mM), bundle E was measured to be ∼20 ± 9.7 MPa, while increasing (Mg^2+^) to 30 and 50 mM displayed a significant reduction in E of bundles, with the lowest E ∼4 ± 1 MPa for 30 mM Mg^2+^ (Figure 4B).

## Discussion

The goal of this study is to investigate the alterations of cation- and depletion-induced actin bundle nanomechanics and organization in the presence of crowding and cations. This work connects how the presence of electrostatic and depletion interactions modulate actin bundle mechanics and organization on the nanoscale. We demonstrate that cation-induced bundles in macromolecular crowding show a reduction in bundle E and an increase in height. Depletion-induced bundles exhibit overall reductions to relative Young’s modulus as well as increase to bundle height with increases in (Mg^2+^). Overall, this study suggests that modulations to bundle mechanics and organization are driven by electrostatic and excluded volume effects measurable on the nanoscale.

For this investigation, we utilized different sizes of crowding agents as well as physiological concentrations of cations that estimated the total volume inside cells (∼80–400 mg/ml) (Zimmerman and Trach, 1991; Rivas et al., 2004; Kuznetsova et al., 2014). The crowded conditions in our experiments were ∼10–200 mg/ml, occupying a significant amount of the available total solution volume previously shown in Romani (2011) and Castaneda *et al*. (2019). The measured nanomechanical properties of bundles in crowding or cation conditions suggest that the bundles may experience changes in interfilament distance, impacting the local deformation of the cantilever tip. Our results demonstrate that cation-induced bundles exhibit a reduction in E with increasing concentrations of Ficoll or PEG (Figures 2A,B). In comparison, Ficoll-induced bundles can sustain their mechanics in cation environments, while PEG bundles are susceptible to cations and undergo alterations to mechanics (Figures 4A,B). Previous investigations to determine the mechanical properties of actin bundles utilized total internal reflection fluorescence (TIRF) microscopy (Castaneda et al., 2018). Although the investigation by Castaneda *et al*. demonstrated that bundle bending persistence length (*L*_p_) can be modulated with varying cation conditions, microscopy imaging is limited by two dimensions (2D), while AFM can perform nanomechanical measurements on the nanoscale and in three dimensions (3D). A recent study utilizing both TIRF and AFM revealed that the nanomechanics of bacteria can withstand greater localized cantilever deformation in 3D rather than longitudinal bending (2D) (Lee, 2018). The elastic response of cation- and depletion-induced bundles exhibited in this study suggests the opposite response to applied external load, potentially due to a change in filament packing that reduces E .

The organization of the filaments (Gov, 2008) and the thickness of bundles (Lieleg et al., 2007) could be key factors in determining bundle nanomechanics—for example, actin bundles induced by depletion interactions were shown to increase in thickness as well as elastic modulus with an increase in the concentrations of PEG (Tharmann et al., 2006). A recent study has shown that crowding can tune the diameter of actin bundles crosslinked by actin binding proteins and possibly impact filament spacing (Park et al., 2021). Divalent cations were previously shown to alter the interfilament distance in bundles, with the greatest filament spacing at ∼7 nm for 30 mM Mg^2+^ (Castaneda et al., 2018). Of note, we observed that the 30 mM Mg^2+^-induced bundles in dilute buffer conditions exhibit striations along the bundle surface; this type of pattern has been previously observed with AFM on purified actin filaments and filaments in cells (Usukura et al., 2016). In addition to bundle organization and interfilament distances, the packing of filaments within the bundle could impact bundle height (Kwon et al., 2006; Gov, 2008). AFM imaging on actin filaments and bundles have demonstrated that the filament height is ∼4 nm and the crosslinked actin bundle height is ∼8 nm (Gilmore et al., 2013). Our results show that the cation-induced bundle height increased up to approximately fourfold with the addition of 10% w/w Ficoll and PEG, with observed bimodal height distributions at 10% w/w PEG (Supplementary Figures S2A–B). The Ficoll-induced bundles maintained their height with minimal changes, while the PEG-induced bundles were shown to display bimodal distributions in the presence of increasing cation conditions (30 mM Mg^2+^) (Supplementary Figures S4A–B). A possible explanation for the changes to the bundle organization and height, as well as bimodal distributions, could be reflected in the opposite dependence of cation and crowder interactions with actin bundles—for instance, Tang *et al*. previously showed that bundles formed through depletion interactions displayed an opposite dependence of cation concentrations, modulating actin bundle formation (Tang et al., 1997). In addition, predictive modeling has suggested that a possible competition can exist between the bending energy of helical filaments and the binding energies of crosslinkers promoting specific bundle sizes (Gov, 2008). Furthermore, Dobramysl *et al*. demonstrated through theoretical modeling that steric effects driven by excluded volume could promote the increase to bundle height and reorganization of filaments within bundles, potentially altering the bundle mechanical properties (Dobramysl et al., 2016). Crowding agent chemical structure, size, and weight could be contributing factors in altering actin bundle organization and height. In our concentration regimes, Ficoll is considered to be a compacted and spherical molecule with a size of ∼40 Å, while PEG 8k is a linear polymer with a size of ∼24 Å (Kuznetsova et al., 2014). Ficoll could promote entropically driven enhanced bundle organization by hindering electrostatic interactions with surrounding filaments (Mardoum et al., 2018). In contrast, the linear crowder PEG could interact with filament domains as previously shown in Castaneda *et al*. (2019). Overall, these changes observed on actin bundle nanomechanics, organization, and geometry driven by crowding and cation interactions can possibly impact the assembly and regulation of actin bundles as well as bundle functions, such as network formation (Miyazaki et al., 2015), mechanosensing (Colombelli et al., 2009), or cell motility (Martiel et al., 2020).

## Conclusion

We have demonstrated through nanoscale imaging and biophysical analysis that cation- and depletion-induced actin bundles can undergo alterations to their nanomechanics and organization by varying macromolecular crowding and cation concentrations. Cation-induced actin bundles experience alterations to both nanomechanics and height. In contrast, Ficoll-induced bundles can sustain their mechanical properties and organization, while PEG-induced bundles are more susceptible to cation environments. Our work leads to the understanding of how actin bundle mechanics and organization are influenced by varying crowding and cations on the nanoscale and bridges the gap in knowledge for determining actin bundle regulatory processes in cells.

## Data Availability

The original contributions presented in the study are included in the article/Supplementary Material. Further inquiries can be directed to the corresponding authors.
