# Supplementary material for: Actin Bundle Nanomechanics and Organization Are Modulated by Macromolecular Crowding and Electrostatic Interactions
Source: Front Mol Biosci. 2021 Nov 26;8:760950. doi: 10.3389/fmolb.2021.760950 (PMC8662701; doi:10.3389/fmolb.2021.760950)
Supplement: Supplementary file 1 [file DataSheet1.DOCX]

**Supplementary Material**

**Actin Bundle Nanomechanics and Organization are Modulated by Macromolecular Crowding and Electrostatic Interactions**

**Nicholas Castaneda^a,b^, Cecile Feuillie^c^, Michael Molinari^c^*, Ellen Hyeran Kang^a,d,e^***

^a^NanoScience Technology Center, University of Central Florida, Orlando, FL 32826, USA

^b^Burnett School of Biomedical Sciences, College of Medicine, University of Central Florida, Orlando, FL 32827, USA

^c^Institute of Chemistry & Biology of Membranes & Nano-objects, CBMN CNRS UMR 5248, IPB, Université de Bordeaux, Pessac, France

^d^Department of Materials Science and Engineering and eDepartment of Physics, University of Central Florida, Orlando, FL 32816, USA

* To whom correspondence should be addressed. Email: Ellen.Kang@ucf.edu and Michael.Molinari@u-bordeaux.fr


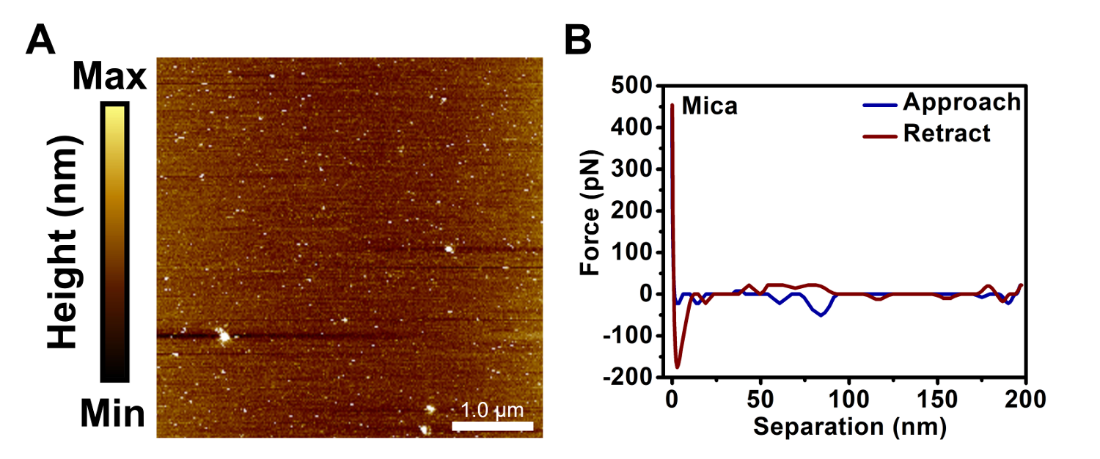


**Figure S1.** AFM imaging and force measurement on mica substrate. (A) Representative image of mica substrate coated with APTES binding agent (0.1% v/v). (B) Representative force curve measurement of mica coated with APTES.


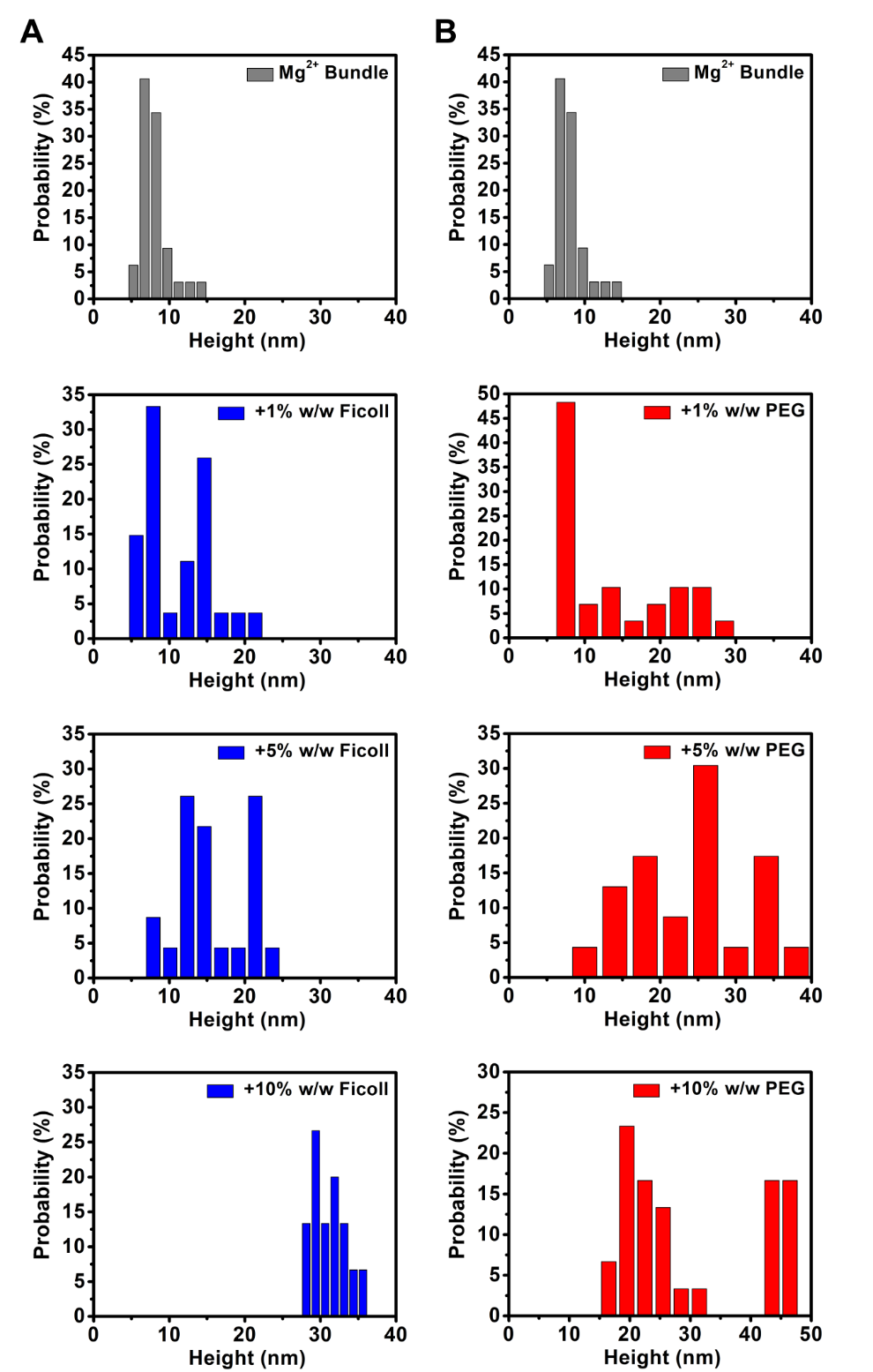


**Figure S2.** Distribution of cation-induced bundle height in various macromolecular crowded environments. Cation-induced actin bundles in 1-10% w/w Ficoll (A) and PEG (B). The number of measurements performed for each of the bundle conditions was ≈ 30.


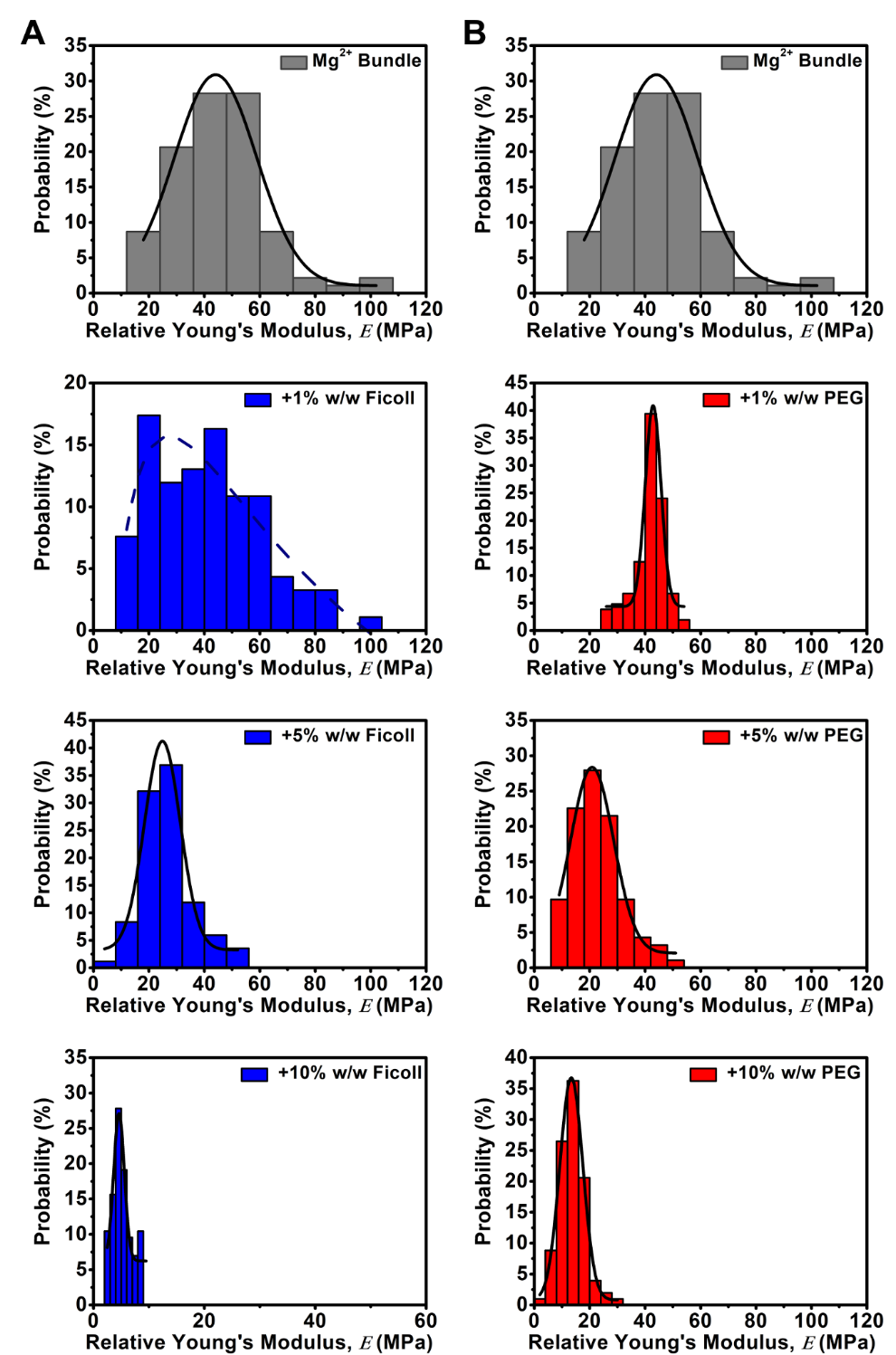


**Figure S3.** Cation-induced bundle Young’s Modulus ($E$) distributions in various macromolecular crowded environments. Cation-induced actin bundles in 1-10% w/w Ficoll (A) and PEG (B). Number of force curves analyzed performed for each of the bundle conditions was N ≈ 150. The line represents bundle $E$ distribution fit with either Gaussian (solid line), log-normal (dashed line), or double exponential (dashed-dot) functions.


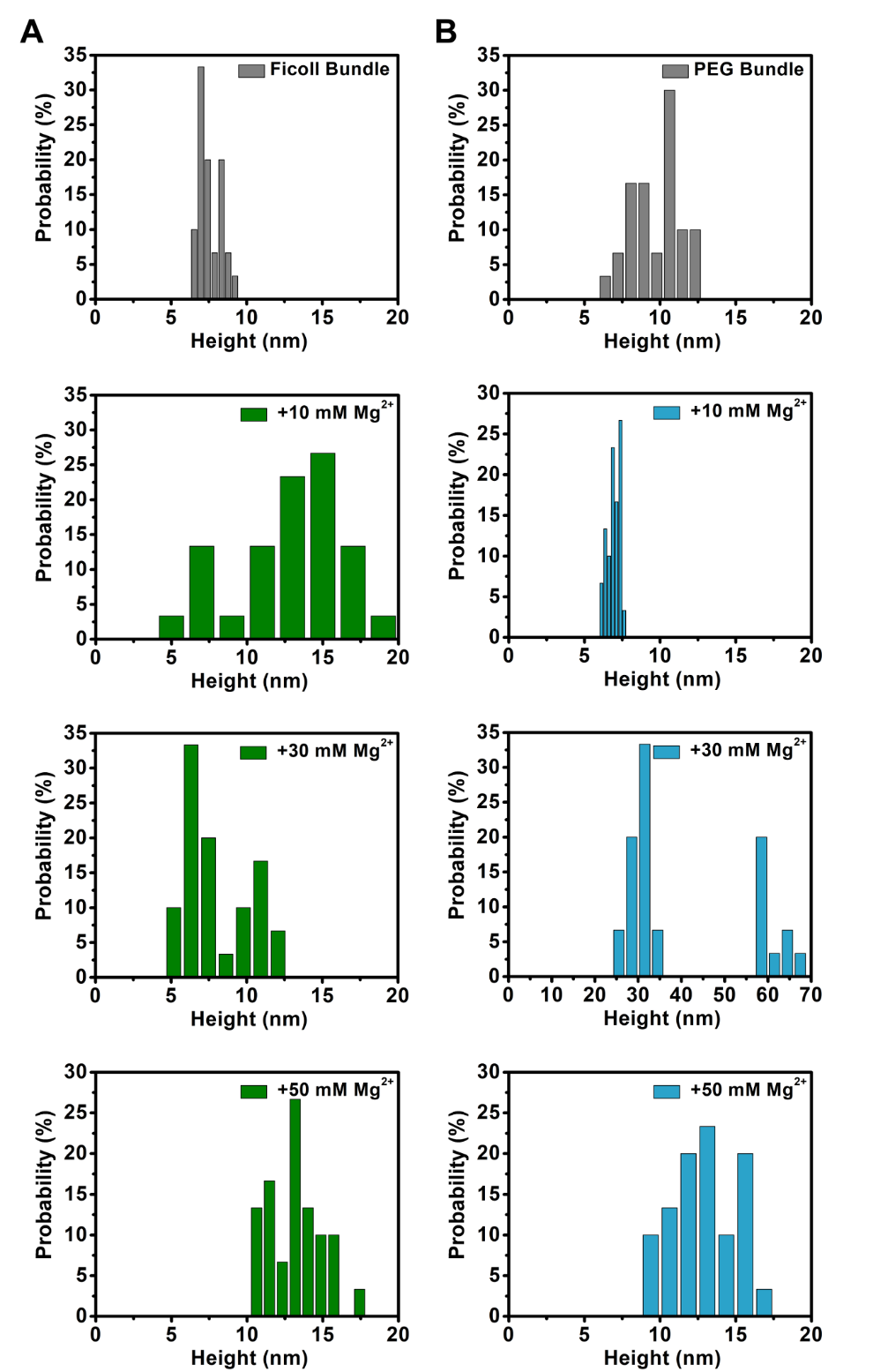


**Figure S4.** Distribution of depletion-induced bundle height in various cation concentrations. (A) Ficoll-induced and (B) PEG-induced actin bundles in 10-50 mM Mg^2+^. The number of measurements performed for each of the bundle conditions was ≈ 30.


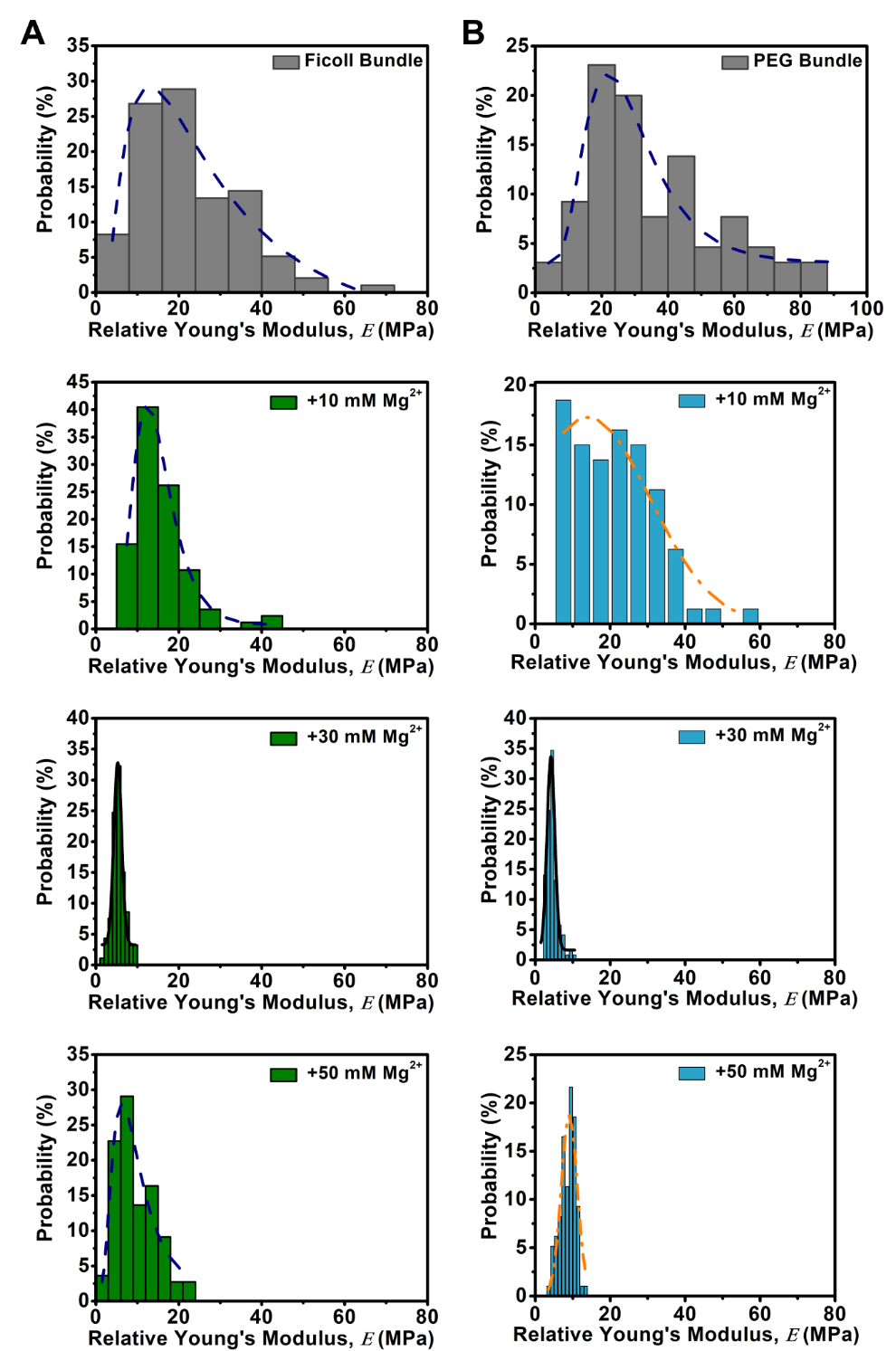


**Figure S5.** Depletion-induced Young’s Modulus ($E$) distributions in various [Mg^2+^]. (A) Ficoll-induced and (B) PEG-induced actin bundles in 10-50 mM Mg^2+^. Number of force curves analyzed performed for each of the bundle conditions was N ≈ 150. The line represents bundle $E$ distribution fit with either Gaussian (solid line), log-normal (dashed line), or double exponential (dashed-dot) functions.
